# Improving Microbial Quality of Ostrich Meat Using Mechanically Deboned Chicken Meat Protein Nanocomposite Coating Incorporated With *Ziziphora clinopodioides* Essential Oil and Eugenol During Cold Storage

**DOI:** 10.1002/fsn3.71759

**Published:** 2026-05-29

**Authors:** Hassan Barkhordari, Majid Aminzare, Hassan Hassanzadazar, Adel Mirza Alizadeh, Mahsa Hashemi, Reza Tahergorabi, Shahin Roohinejad

**Affiliations:** ^1^ Zanjan Pharmaceutical Biotechnology Research Center Zanjan University of Medical Sciences Zanjan Iran; ^2^ Department of Food Safety and Hygiene, School of Public Health Zanjan University of Medical Sciences Zanjan Iran; ^3^ Nutrition and Food Security Research Center Zanjan University of Medical Sciences Zanjan Iran; ^4^ Food and Nutritional Sciences Program North Carolina Agricultural and Technical State University Greensboro North Carolina USA; ^5^ Division of Food and Nutrition, Burn and Wound Healing Research Center Shiraz University of Medical Sciences Shiraz Iran

**Keywords:** eugenol, mechanically deboned meat protein, microbial stability, nanocomposite, ostrich meat, sensory properties, *Ziziphora clinopodioides* essential oil

## Abstract

The objective of this study was to compare the in vitro antibacterial activities of conventional (non‐nanocomposite) and nanocomposite (nanoclay‐based) coatings made from mechanically deboned chicken meat protein (MDCM‐P) enriched with *Ziziphora clinopodioides* essential oil (ZEO) and/or eugenol (EUG) against foodborne pathogens. Subsequently, best‐performing coatings identified through in vitro tests were evaluated for effects on microbial quality, safety (via inoculation with *Staphylococcus aureus* and *Escherichia coli* O157:H7), and sensory characteristics of ostrich meat during 21‐day storage at 4°C. Based on GC–MS results, carvacrol (62.5%) was the major compound of ZEO. Particle size of nanocomposites ranged from 137 to 514.8 nm. The well‐diffusion method showed that *Listeria monocytogenes* was the most sensitive and *E. coli* O157:H7 the most resistant bacterium. Ostrich meat coated with MDCM‐P nanocomposite containing ZEO (16 mg/mL) and EUG (16 mg/mL) demonstrated improved microbial quality compared to uncoated meat, showing reductions in total viable bacteria (9.29 to 6.33 log_10_ CFU/g), psychrotrophs (10.41 to 6.45 log_10_ CFU/g), lactic acid bacteria (8.36 to 6.07 log_10_ CFU/g), coliforms (8.41 to 5.46 log_10_ CFU/g), and molds and yeasts (8.17 to 5.26 log_10_ CFU/g), and enhanced microbial safety by lowering *S. aureus* (7.44 to 5.27 log_10_ CFU/g) and *E. coli* O157:H7 (8.42 to 5.25 log_10_ CFU/g) counts. Furthermore, it exhibited improved sensory attributes, achieving an overall acceptability score of 5.4 compared to 2.15 for control. These findings suggest that MDCM‐P nanocomposite coating containing ZEO and EUG shows potential as an active packaging material to improve microbial quality and safety of refrigerated ostrich meat.

## Graphical abstract

Nanocomposite coatings based on mechanically deboned chicken meat protein incorporated with nanoclay, *Ziziphora clinopodioides* essential oil, and eugenol effectively improved the microbial quality, safety, and sensory attributes of refrigerated ostrich meat.

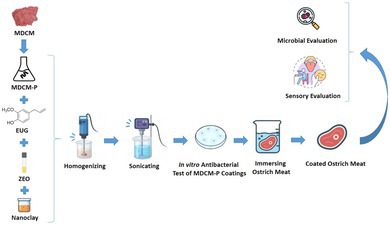

## 1 Introduction

Ostrich meat is promoted as a healthy red meat because of its high unsaturated fatty acid content and lower sodium, saturated fat, cholesterol, and intramuscular fat levels. It is therefore suitable for individuals pursuing a healthier lifestyle. However, given the significant global demand for ostrich meat and its high pH, vitamins, iron, and protein levels that promote microorganism growth, ensuring its microbiological standards is crucial (Heydari et al. 2020; Kiakojori et al. 2024). Meat can harbor significant numbers of pathogenic and spoilage‐related microorganisms during slaughtering and subsequent processing stages. During refrigerated storage, the microbial population on the surface of fresh meat typically evolves. When meat spoilage‐related microorganisms exceed a specific threshold, visible signs such as off‐odors and surface slime appear. Additionally, common pathogens such as *Staphylococcus aureus* and *Escherichia coli* O157:H7, often linked to foodborne outbreaks, are frequently found in meat. Contamination with *E. coli* O157:H7 typically arises from sewage, contaminated hides, or equipment during slaughter, whereas *S. aureus* is introduced from sources such as skin, equipment, or infected personnel. These pathogens can cause self‐limiting gastrointestinal infections or severe, potentially fatal conditions, particularly in immunocompromised individuals, the elderly, and young children (Khezrian and Shahbazi 2018; Saricaoglu and Turhan 2019).

Applying natural antimicrobial substances with biodegradable films and coatings is a promising method to inhibit microbial growth in fresh meat (Kiakojori et al. 2024). This approach has gained attention due to health concerns associated with synthetic preservatives (Heydari et al. 2020). In this regard, plant‐derived antimicrobials, including essential oils (EOs) and their bioactive components, present a promising option. However, their application is often constrained by flavor‐related issues that can lead to undesirable organoleptic properties in foods. To overcome this challenge, combining these preservatives has emerged as an effective strategy to reduce adverse sensory impacts (Khezrian and Shahbazi 2018). *Ziziphora clinopodioides*, a member of the Lamiaceae family, is extensively cultivated across various regions worldwide, particularly in the Middle East, and is valued in Persian healing practices for its broad spectrum of therapeutic benefits. The *Z. clinopodioides* essential oil (ZEO) has also been utilized to improve the microbial quality, safety, and sensory characteristics of different types of meat under refrigeration (Hasan et al. 2019; Shahbazi et al. 2016; Shavisi, Khanjari, et al. 2017). Similarly, eugenol (EUG), an aromatic phenolic compound derived from plants like cloves and cinnamon, exhibits notable antimicrobial properties in muscle foods (Yousefizadeh et al. 2022; Zhao et al. 2022).

Amid growing concerns about plastic pollution and the harmful effects of packaging residues, recent efforts have focused on developing biodegradable edible polymers for food packaging (Heydari et al. 2020). Proteins derived from low‐cost agricultural residues, including mechanically deboned chicken meat (MDCM: a poultry industry by‐product), can serve as biopolymer sources for edible films and coatings. However, MDCM‐protein (MDCM‐P) films have limited mechanical and functional properties, restricting their use as packaging materials (Saricaoglu and Turhan 2020). It has been proven that incorporating EOs and their bioactive compounds into MDMC‐P polymer can increase its functional properties and antimicrobial activity (Saricaoglu and Turhan 2019, 2020). Additionally, employing nanocomposite technology by incorporating nanoclay, can significantly enhance these properties in MDCM‐P‐based food packaging systems (Menezes et al. 2017). Polymer nanocomposites are mainly created by dispersing a nanofiller (such as nanoclay) into a polymer matrix, thereby improving the mechanical features of edible biopolymers used in food packaging. Active nanocomposites can enhance microbial safety and quality for packaged foods by releasing plant‐derived bioactive compounds and blocking the penetration of water and oxygen. In food packaging, nanoclay is extensively used as a nanofiller, valued for its affordability, accessibility, and biocompatibility, while also boosting the antimicrobial properties of packaging materials (Mozafar et al. 2025; Nath et al. 2022).

Numerous studies have investigated the effects of edible food packaging systems comprising ZEO or EUG individually on the microbial and sensory quality of muscle foods (Hasan et al. 2019; Yousefizadeh et al. 2022; Zhao et al. 2022). However, research on the antimicrobial features of MDCM‐P films and coatings enriched with EOs, both in vitro and actual food systems, remains limited (Saricaoglu and Turhan 2019, 2020). To our knowledge, no comparative study has investigated the in vitro antibacterial interactions between ZEO and EUG in conventional (non‐nanocomposite) and nanocomposite edible films or coatings. In addition, their combined antimicrobial and sensory effects on meat products have not yet been evaluated. Therefore, this study aimed to: (1) Prepare ZEO and analyze its chemical composition; (2) Develop and characterize conventional and nanocomposite (incorporating nanoclay) MDCM‐P coatings with ZEO and/or EUG; (3) Assess the in vitro antibacterial effects of these coatings against four foodborne pathogens and determine the antibacterial interactions between ZEO and EUG; (4) Evaluate the microbial quality, safety (via *S. aureus* and *E. coli* O157:H7 inoculation), and sensory properties of ostrich meat coated with the most effective formulations during 21 days of refrigerated storage (4°C).

## 2 Materials and Methods

### 2.1 Material

Fresh ostrich meat and *Z. clinopodioides* plant were obtained directly from local stores in Zanjan City, Iran. MDCM was also obtained from DARA Meat Product Company, Shahriar, Iran. Eugenol, nanoclay powder (hydrophilic bentonite, montmorillonite), and other chemicals such as ethanol 70%, Tween 80, anhydrous sodium sulfate, and glycerol were supplied by Sigma‐Aldrich (St. Louis, MO, USA). Microbial culture media including Peptone Water (PW), de Man‐Rogosa and Sharpe (MRS) agar, Potato Dextrose Agar (PDA), Plate Count Agar (PCA), Mueller‐Hinton Agar (MHA), Mannitol Salt Agar (MSA), Eosin Methylene Blue (EMB) agar, and Violet Red Bile Agar (VRBA) were purchased from Merck (Darmstadt, Germany). Lyophilized microbial cultures of *Escherichia coli* O157:H7 (PTCC 1860), *Staphylococcus aureus* (PTCC 1431), *Salmonella enteritidis* ( *Salmonella enterica* subsp. *enterica* serovar Enteritidis: PTCC 1787), and *Listeria monocytogenes* (PTCC 1783) were sourced from the center of industrial microorganisms, Iranian Research Organization for Science and Technology (IROST).

### 2.2 ZEO Extraction

*Z. clinopodioides* leaves were washed thoroughly and dried at 25°C for 14 days. The dried leaves were crushed by a blender (Pars Khazar, Tehran, Iran). A 100 g of it was placed in the Clevenger‐type distillation container (KOL, Behr, Düsseldorf, Germany), and the volume was adjusted to 1000 mL with deionized water. The mixture was then subjected to distillation at 100°C for 3.5 h. This method was repeated about 20 times to obtain about 10 mL of ZEO. Anhydrous sodium sulfate was used to dehydrate the extracted ZEO during the night, after which it was stored in dark glass containers at 4°C until further application (Shahbazi et al. 2016).

### 2.3 Gas Chromatography–Mass Spectrometry Analysis of ZEO

For the GC–MS analysis, a Hewlett Packard 5890 Series II gas chromatograph coupled to an HP 5972 mass selective detector was used. The system was equipped with a DB‐5 capillary column (30 m long, an inner diameter of 0.25 mm, and 0.25 μm in film thickness). The temperature ranged from 50°C to 265°C with a gradient of 2.5°C/min. The injector temperature was set at 250°C, and the detector operated with an ionization energy of 70 eV. Helium was used as the carrier gas at a flow rate of 1.2 mL/min, with a split ratio of 20:1. The chemical compounds of ZEO were identified by comparing their retention parameters with available data and typical mass spectral fragmentation profiles (Wiley/NBS) as well as with the database of the National Institute of Standards and Technology (NIST) (Shahbazi et al. 2016).

### 2.4 MDCM‐P Extraction

The MDCM was first defatted using diethyl ether in a mixer overnight. The fat‐extracted MDCM was then combined with water in a 1:9 (w/v) proportion for 2 min using a mixer (HB‐MQ 500 Soup, BRAUN, Leipzig, Germany). To solubilize the protein, the solution's pH level was adjusted to 12 with 5 M NaOH, followed by centrifugation at 9000× *g* for 30 min at 4°C, after which the liquid phase was collected. Protein precipitation was achieved by adjusting the pH to 4.7 using 5 M HCl and centrifuging again at 9000× *g* for 10 min at 4°C. The supernatant was discarded, and the precipitated protein was dried at 40°C for 24 h. The resulting MDCM‐P was ground and kept in a glass jar at 4°C until further use (Saricaoglu et al. 2017).

### 2.5 Preparing Conventional and Nanocomposite MDCM‐P Coatings Containing ZEO and/or EUG

Two types of coatings were developed in this study: a conventional MDCM‐P coating and a nanocomposite coating obtained by incorporating nanoclay into the MDCM‐P matrix.

To prepare conventional coatings, 4 g of MDCM‐P powder was stirred in 100 mL of deionized water using a homogenizer (Silent Crusher M, Heidolph, Schwabach, Germany) for 1 min at 14,000 rpm, and NaOH (5 M) was used to adjust the pH to 11.5 for better solubility. After that, 40% glycerol (v/v) was added to the coating‐forming solution (CFS) as a plasticizer. Then, the solution was blended for 2 min at 5000 rpm and was incubated in a water bath at 90°C for 1 h. The temperature of the mixture was then reduced to 25°C. Considering the findings from the preliminary evaluations, different concentrations of ZEO and EUG (0, 1, 2, 4, 8, and 16 mg/mL) previously mixed with Tween 80 (0.2 w/w of ZEO or EUG) were added separately to the CFSs. Also, the two concentrations of ZEO and EUG that exhibited the best results were added in combination with the CFSs. Then, the CFSs were homogenized at 12000 rpm for 5 min at 25°C (Saricaoglu and Turhan 2019).

For the nanocomposite system, nanoclay (3% w/w relative to MDCM‐P) was incorporated into the already‐prepared MDCM‐P CFSs and mixed using a blender at 5000 rpm for 10 min to obtain a reinforced polymer matrix. Subsequently, ZEO, EUG, and their mixture, along with Tween 80, were incorporated into the cooled CFSs at the same concentrations as the conventional coating. The resulting composition was further homogenized at 20,000 rpm for 5 min. Sonication (700 W, 20 kHz, 5 min, probe depth 20 mm) was applied at 25°C to reduce particle size, while samples were kept in cold water to prevent overheating (Mozafar et al. 2025). In this system, nanoclay acted as a nanoscale reinforcing filler dispersed within the MDCM‐P polymer matrix, while sonication was applied to enhance nanoclay dispersion and reduce droplet size to the nanoscale range. The studied treatments are summarized in Table 1.

**TABLE 1:** Different coatings and their abbreviations.

No.	Treatment	Description
1	MDM	Conventional mechanically deboned chicken meat protein coating
2	MDM‐ZEO	Conventional mechanically deboned chicken meat protein coating containing different concentrations of *Ziziphora clinopodioides* essential oil
3	MDM‐EUG	Conventional mechanically deboned chicken meat protein coating containing different concentrations of Eugenol
4	MDM‐ZEO + EUG	Conventional mechanically deboned chicken meat protein coating containing different concentrations of *Ziziphora clinopodioides* essential oil and Eugenol
5	NMDM	Nanocomposite mechanically deboned chicken meat protein/nanoclay coating
6	NMDM‐ZEO	Nanocomposite mechanically deboned chicken meat protein/nanoclay coating containing different concentrations of *Ziziphora clinopodioides* essential oil
7	NMDM‐EUG	Nanocomposite mechanically deboned chicken meat protein/nanoclay coating containing different concentrations of Eugenol
8	NMDM‐ZEO + EUG	Nanocomposite mechanically deboned chicken meat protein/nanoclay coating containing different concentrations of *Ziziphora clinopodioides* essential oil and Eugenol

### 2.6 Polydispersity Index and Particle Size of MDCM‐P Nanocomposite Coatings

The polydispersity index (PDI) and average particle size were measured on freshly prepared CFSs of the nanocomposite formulations using dynamic light scattering (DLS) technique with a Zetasizer device (Malvern Panalytical Ltd., Malvern, UK) (Mozafar et al. 2025).

### 2.7 In Vitro Antibacterial Activities of Conventional and Nanocomposite MDCM‐P Coatings Containing ZEO and/or EUG

#### 2.7.1 Bacterial Strain Preparation

The antibacterial efficacy of conventional and nanocomposite MDCM‐P coatings was evaluated against two gram‐negative bacterial strains ( *S. enteritidis* and *E. coli* O157:H7) and two gram‐positive strains ( *L. monocytogenes* and *S. aureus* ). The optical density (OD) technique was used to make and quantify bacterial inoculations. Freshly cultured bacterial colonies were diluted in 10 mL of PW (0.1% w/v), and their absorbance was measured at 600 nm using a spectrophotometer device (DR 5000, HACH, Düsseldorf, Germany) to modify turbidity to a 0.5 McFarland standard (~1–2 × 10^8^ CFU/mL). These suspensions were further diluted in PW (0.1% w/v) to achieve ~1–2 × 10^6^ CFU/mL and confirmed by counting the bacterial on PCA at 37°C for 24 h (Mozafar et al. 2025).

#### 2.7.2 Well‐Diffusion Method

Bacterial suspensions (~1–2 × 10^6^ CFU/mL) were grown on MHA medium according to the spread plate method. Wells (6 mm diameter) were made on the surface of the inoculated agar and 20 μL of freshly prepared conventional and nanocomposite CFSs containing different concentrations of antimicrobial agents were added. Finally, the agar culture plates were maintained at 37°C for 24 h. Chloramphenicol disk (30 μg/disk) was used as a positive standard. Inhibition zones were measured by calipers and reported in mm (Mozafar et al. 2025). The antibacterial effectiveness of the coatings was categorized as follows (Aminzare et al. 2022):

- Weak antibacterial activity: Inhibitory zone ≤ 12 mm;
- Moderate antibacterial activity: Inhibitory zone between 12 and 20 mm;
- Strong antibacterial activity: Inhibitory zone ≥ 20 mm.

### 2.8 Applying Conventional and Nanocomposite MDCM‐P Coatings Containing ZEO and EUG on Ostrich Meat

#### 2.8.1 Ostrich Meat Preparation

Fresh ostrich meat was promptly delivered to the laboratory in a polystyrene container with ice packs in hygienic settings and subsequently cut into 25 g pieces. According to the findings from the in vitro analysis, conventional and nanocomposite coatings with optimal concentrations of ZEO and EUG were selected to coat the ostrich meats. Therefore, four groups were recognized for this study:

1. CON (Control): Uncoated ostrich meat sample;
2. NMDM: Ostrich meat sample treated with nanocomposite MDCM‐P/nanoclay coating without ZEO and EUG;
3. MDM‐ZEO + EUG: Ostrich meat sample treated with conventional MDCM‐P coating containing ZEO (16 mg/mL) and EUG (16 mg/mL);
4. NMDM‐ZEO + EUG: Ostrich meat sample treated with nanocomposite MDCM‐P/nanoclay coating containing ZEO (16 mg/mL) and EUG (16 mg/mL).

The samples were soaked in their respective coating treatments for 3 min and allowed to dry for 10 min under a laminar hood at 25°C. They were then stored in plastic bags at 4°C and subjected to various tests at intervals of 0, 7, 14, and 21 days.

#### 2.8.2 Microbial Quality Evaluation

Briefly, 25 g of ostrich meat samples were transferred to sterilized stomacher bags with 225 mL of PW (0.1% v/w) and blended with a stomacher (Seward Ltd., London, UK) at 400 strokes/min at 25°C for 1 min. Serial dilutions were prepared, and 10 μL of each dilution was spread onto specific agar plates using the drop plate method. For psychrotrophic count (PTC) and total viable count (TVC), samples were cultured on PCA media and incubated at 7°C for 10 days and 37°C for 24 h, respectively. Lactic acid bacteria (LAB) were assessed using MRS agar, incubated at 25°C for 5 days under microaerophilic settings (anaerobic container with a GasPak system type C). Coliform bacteria were enumerated on VRBA after incubation at 37°C for 24 h. The spread plate method was used to count molds and yeasts (M&Y), and colonies were counted on PDA after 5 days of growth at 25°C. The results were reported as log_10_ CFU/g of the meat sample (Heydari et al. 2020).

#### 2.8.3 Microbial Safety Evaluation

The microbial safety of ostrich meat samples was assessed by inoculating them with *S. aureus* and *E. coli* O157:H7 bacteria, following the method of Heydari et al. (2020) with modifications. Each 25 g piece of ostrich meat was treated with 70% (v/v) ethanol to eliminate contamination. Sterilized samples were inoculated with approximately 1–2 × 10^5^ CFU/g of each pathogen, dipped in coating solutions, and kept in sterilized polyethylene bags at 4°C for 21 days. At specified intervals, 25 g of each sample was homogenized with 225 mL of peptone water (0.1% w/v) using a stomacher, serially diluted, and cultured on MSA and EMB agar plates at 37°C for 24 h using the drop plate method to determine *S. aureus* and *E. coli* O157:H7 counts, respectively. The findings were expressed as log_10_ CFU/g of the meat sample.

#### 2.8.4 Sensory Evaluation

Ten semi‐trained panelists (5 male and 5 female non‐smokers from Zanjan University of Medical Sciences) were selected based on their performance in a pre‐test to evaluate the sensory features of ostrich meat. Introductory meetings were held before testing to ensure panelists understood and agreed on the sensory properties to be assessed. The fillets were prepared in a microwave (SolarDOM SD‐3853WCR, LG, Seoul, South Korea) following the instructions from the manufacturer. Sensory attributes were assessed using a 9‐point scale (1: intolerable, 9: excellent). The assessments were conducted in private booths under incandescent lighting, with panelists cleansing their palates between tests using salt‐free crackers and 25°C water. Samples were coded at random and assessed semi‐blindly. Based on prior studies, samples scoring above 4 were deemed acceptable. Color, odor, and overall acceptability were estimated on days 0, 7, 14, and 21, while taste was assessed on days 0 and 7 only. Informed consent was obtained from all participants, and the study received approval from the Ethics Committee of Zanjan University of Medical Sciences (Ethics Code: IR.ZUMS.BLC.1401.053) (Heydari et al. 2020).

### 2.9 Statistical Analysis

Descriptive statistics were reported as “Mean ± SE,” and the analyses were performed using IBM‐SPSS Statistics (Version 26 for Windows; IBM Inc.). To measure the PDI and particle size of nanocomposites as well as in vitro antibacterial assessments of coatings, three independent batches were prepared for each type of coating and a random selection from each batch was conducted. The results were then compared using a one‐way ANOVA and subsequently performing Tukey's honestly significant difference post hoc test (*α* = 0.05).

For the food model study, four experimental groups were established: (1) CON, (2) NMDM, (3) MDM‐ZEO + EUG, and (4) NMDM‐ZEO + EUG. Three independent batches were prepared for each group, and samples were randomly chosen at each of the four time points (0, 7, 14, and 21 days). Microbiological analysis was performed separately for each sample. These tests utilized Univariate Analysis of Variance, with treatments and storage time treated as fixed factors in the statistical model, and their interaction was also examined. In sensory evaluation, treatments and time intervals were considered as fixed factors, whereas the group of panelists was considered as a random factor. The interaction between fixed and random factors was included in the model. Tukey's post hoc test was conducted to identify significant differences (*α* = 0.05). All charts were generated using Microsoft Excel 2013.

## 3 Results and Discussion

### 3.1 ZEO Chemical Composition

The relative percentages of the chemical constituents of ZEO are listed in Table 2. According to the results, GC–MS analysis identified twenty‐three distinct chemical compounds, which collectively constitute 99.24% of the total EO content. The most abundant constituents were carvacrol (62.5%) and thymol (19.5%), together making up 82% of the identified compounds, which is consistent with previous studies (Shahbazi and Shavisi 2016). Additionally, p‐Cymene, 1,8‐Cineole, and Terpinene each contributed approximately 4% to the ZEO composition, while the remaining compounds were present at concentrations below 1%. In contrast to our results, the main compounds identified by Hasan et al. (2019) in ZEO were geraniol (20.62%) and carvacrol (18.17%). Variations in the ZEO components reported in different studies may be due to botanical variations, growing place, growth stage, genetic characteristics, geographical conditions, climate, seasonal changes, and the methods used to prepare the EO (Hasan et al. 2019; Shahbazi and Shavisi 2016).

**TABLE 2:** Chemical composition of *Ziziphora clinopodioides* essential oil.

No.	Compound name	Area (%)	KI	RT
1	α‐Pinene	0.2	940	11.43
2	Camphene	0.26	960	11.80
3	Sabinene	0.41	972	11.30
4	β‐Pinene	0.3	982	11.45
5	Β‐Myrcene	0.36	997	12.61
6	α‐Terpinene	0.44	1016	15.10
7	p‐Cymene	4.02	1027	16.43
8	Limonene	0.80	1034	16.70
9	1,8‐Cineol	4.10	1038	15.31
10	Terpinene	4.09	1030	18.31
11	linalool	0.16	1105	21.5
12	borneol	0.5	1142	24.40
13	L‐Menthone	0.7	1180	25.30
14	Eugenol	0.28	1230	26.70
15	α‐Ylangene	0.1	1243	27.30
16	Bourbonen	0.1	1260	28.21
17	thymol	19.5	1293	29.61
18	carvacrol	62.5	1291	30.57
19	β‐Selinene	0.21	1487	33.32
20	δ‐Selinen	0.13	1502	34.20
21	Spathulenol	0.32	1573	35.20
22	Caryophyllene oxide	0.27	1585	40.23
23	β‐Selinene	0.21	1590	42.10
Total		99.24		

Abbreviations: KI, Kovats indices; RT, retention time.

### 3.2 Polydispersity Index and Particle Size of Nanocomposite Coatings

Nanocomposite particle size, referring specifically to the droplet size of the coating‐forming solutions (CFSs), was evaluated because this parameter strongly influences the stability, physicochemical behavior, and loading efficiency of bioactive compounds (Mozafar et al. 2025). Table 3 presents the PDI and mean droplet size of nanocomposite MDCM‐P CFSs containing different concentrations of ZEO and/or EUG. Generally, an increase in ZEO or EUG concentrations resulted in larger particle sizes (*p* ≤ 0.05), aligning with Mozafar et al. (2025), who reported similar findings in starch nanocomposite coatings with varying concentrations of *Thymus fedtschenkoi* Ronniger EO and thymoquinone. This increase in particle diameter is due to the development of a dense, thick layer of biopolymers around the EO droplets. Additionally, the incorporation of ZEO and EUG into the multilayer structure of nanoclay particles plays a major role in determining particle size. The size and stability of the nanoparticles are affected by elements such as the type and concentration of surfactants and EO, solution viscosity, and base polarity. Furthermore, by generating interfacial waves and microbubbles, ultrasound treatment effectively reduces the average droplet size and PDI, facilitating the breakdown of oil particles (Mozafar et al. 2025). The PDI reflects the disparity in the particle distribution, where lower values indicate greater stability and uniformity of particles. As shown in Table 3, the high PDI value observed for NMDM (0.929 ± 0.043) is likely attributed to incomplete dispersion and partial aggregation of protein‐nanoclay structures, resulting in broad and multimodal droplet‐size distributions (de Carvalho‐Guimarães et al. 2022; Ganley and Van Duijneveldt 2016). After incorporation of low concentrations of ZEO and/or EUG together with Tween‐80 and high‐energy homogenization/sonication, the PDI value decreased significantly (*p* ≤ 0.05). This phenomenon can be attributed to the formation of a more uniform droplet population due to improved steric and interfacial stabilization. Previous studies have shown that the combination of a non‐ionic surfactant (e.g., Tween‐80) with ultrasonic emulsification can markedly reduce both droplet size and PDI in protein‐based nanoemulsions, even in the presence of hydrophobic oils. Therefore, the sudden reduction in PDI and droplet size at low ZEO and/or EUG concentrations is attributed to an optimal surfactant‐to‐oil ratio combined with sonication and nanoclay stabilization (Gomes et al. 2022; Gul et al. 2018; Modarres‐Gheisari et al. 2019).

**TABLE 3:** Particle size and polydispersity index of nanocomposite mechanically deboned chicken meat protein/nanoclay coating‐forming solutions containing different concentrations of ZEO and/or EUG (Mean ± SE).

Sample	Concentration (mg/mL)	Particle size (nm)	Polydispersity index
NMDM	—	514.8 ± 14.7^a^	0.929 ± 0.043^a^
NMDM‐ZEO	1	137.2 ± 5.6^g^	0.193 ± 0.068^e^
	2	143.2 ± 8.7^g^	0.270 ± 0.085^cde^
	4	160.3 ± 8.3^g^	0.282 ± 0.101^cde^
	8	273.1 ± 2.3^e^	0.385 ± 0.055^bcde^
	16	334.4 ± 10.5^bcd^	0.532 ± 0.174^bc^
NMDM‐EUG	1	148.8 ± 9.2^g^	0.221 ± 0.086^e^
	2	162.3 ± 10.5^g^	0.283 ± 0.080^cde^
	4	212.6 ± 8.7^f^	0.290 ± 0.114^cde^
	8	343.5 ± 8.5^bc^	0.423 ± 0.138^bcde^
	16	374.3 ± 11.3^b^	0.580 ± 0.094^b^
NMDM‐ZEO + EUG	ZEO (8) + EUG (8)	290.7 ± 3.2^de^	0.416 ± 0.300^bcde^
	ZEO (8) + EUG (16)	310.3 ± 4.0^cde^	0.445 ± 0.098^bcde^
	ZEO (16) + EUG (8)	340.7 ± 12.4^bc^	0.487 ± 0.035^bcd^
	ZEO (16) + EUG (16)	363.3 ± 10.4^b^	0.570 ± 0.059^b^

*Note:* Values marked by different letters within the same columns are significantly different according to Tukey's test (*p* ≤ 0.05).

Abbreviations: NMDM, nanocomposite mechanically deboned chicken meat protein/nanoclay coating‐forming solution; NMDM‐EUG, nanocomposite mechanically deboned chicken meat protein/nanoclay coating‐forming solution containing eugenol; NMDM‐ZEO, nanocomposite mechanically deboned chicken meat protein/nanoclay coating‐forming solution containing *Z. clinopodioides* essential oil; NMDM‐ZEO + EUG, nanocomposite mechanically deboned chicken meat protein/nanoclay coating‐forming solution containing *Z. clinopodioides* essential oil and eugenol.

However, at higher ZEO and/or EUG concentrations, the PDI values increased again. This behavior is attributed to the hydrophobic nature of EOs and the saturation of the stabilizing capacity of the surfactant, both of which promote droplet aggregation at elevated EO concentrations. This increases the likelihood of droplet collision and coagulation, ultimately leading to a broader size distribution and greater heterogeneity within the nanocomposite matrix (Kalateh‐Seifari et al. 2021; McClements 2016). A similar trend was also reported by Mozafar et al. (2025), who observed that increasing the concentration of EO components in starch–nanoclay nanocomposite coatings containing *Thymus fedtschenkoi* Ronniger EO and/or thymoquinone led to higher PDI values due to enhanced droplet aggregation and reduced system stability.

### 3.3 In Vitro Antibacterial Activities of Conventional and Nanocomposite MDCM‐P Coatings

Table 4 illustrates the microbial growth inhibition areas produced by conventional and nanocomposite MDCM‐P coatings, which incorporate varying concentrations of ZEO or EUG, against four foodborne pathogenic bacteria, using the well‐diffusion technique. According to the results, MDM and NMDM coatings without ZEO and EUG showed no inhibition zones against the studied bacteria, which is consistent with Saricaoglu and Turhan (2020), who similarly reported that MDCM‐P film without EOs exhibits no antimicrobial activity. Also, the antimicrobial features of the coatings were significantly influenced by the concentration of ZEO or EUG (*p* ≤ 0.05). MDM and NMDM coatings containing 16 mg/mL ZEO or EUG against all tested bacteria, and NMDM coatings containing 8 mg/mL ZEO or EUG against *L. monocytogenes* , showed moderate antimicrobial effects (12 < inhibition zone < 20 mm). However, other coatings showed weak antimicrobial effects (inhibition zone ≤ 12 mm). The antibacterial effects of ZEO are due to its primary phenolic compounds, particularly oxygenated monoterpenes such as carvacrol and thymol. These compounds exhibit potent antibacterial activity primarily due to the acidic behavior of the hydroxyl groups and their participation in hydrogen bond development (Shahbazi and Shavisi 2016). Carvacrol and thymol in ZEO enhance the distribution of electrons across double bonds and ATP in bacteria through proton release, leading to the disruption of the bacterial wall (Naeeji et al. 2020). The main antibacterial mechanism of EUG is linked to its capacity to enhance the permeability of the bacterial cytoplasmic membrane, ultimately leading to the disturbance of the cell wall (Aminzare et al. 2022). Saricaoglu and Turhan (2020) attributed the in vitro antimicrobial efficacy of MDCM‐P films with thyme and clove EOs to their main components: thymol and carvacrol in thyme, and EUG in clove. Consistent with our findings, Naeeji et al. (2020) demonstrated the in vitro antimicrobial activity of basil seed mucilage–chitosan films containing ZEO, and Aminzare et al. (2022) similarly reported in vitro antibacterial properties of carboxymethyl cellulose films incorporated with EUG against foodborne pathogens.

**TABLE 4:** Antibacterial activity of conventional and nanocomposite mechanically deboned chicken meat protein/nanoclay coating‐forming solutions containing ZEO or EUG using well‐diffusion method (Mean ± SE).

Sample	Concentration (mg/mL)	Inhibition zones (mm)			
		*E. coli* O157:H7	*S. enteritidis*	*S. aureus*	*L. monocytogenes*
MDM‐ZEO	0	ND	ND	ND	ND
	1	1.33 ± 0.33^a^ (+)	2.00 ± 0.00^a^ (+)	2.67 ± 0.33^a^ (+)	3.00 ± 0.58^a^ (+)
	2	2.00 ± 0.00^ab^ (+)	2.67 ± 0.33^ab^ (+)	3.00 ± 0.58^a^ (+)	3.33 ± 0.33^a^ (+)
	4	2.33 ± 0.33^ab^ (+)	3.00 ± 0.00^ab^ (+)	3.33 ± 0.33^a^ (+)	5.00 ± 0.58^a^ (+)
	8	6.67 ± 0.33^c^ (+)	7.67 ± 0.33^d^ (+)	8.00 ± 0.58^b^ (+)	12.00 ± 0.00^c^ (+)
	16	11.00 ± 0.58^d^ (+)	15.33 ± 0.88^e^ (++)	16.00 ± 0.58^d^ (++)	17.67 ± 0.33^de^ (++)
NMDM‐ZEO	0	ND	ND	ND	ND
	1	2.33 ± 0.33^ab^ (+)	3.00 ± 0.00^ab^ (+)	3.67 ± 0.33^a^ (+)	4.00 ± 0.00^a^ (+)
	2	3.00 ± 0.00^ab^ (+)	4.33 ± 0.33^bc^ (+)	4.67 ± 0.67^a^ (+)	4.67 ± 0.88^a^ (+)
	4	3.67 ± 0.33^b^ (+)	5.67 ± 0.67^c^ (+)	7.33 ± 0.67^b^ (+)	8.33 ± 0.33^b^ (+)
	8	8.00 ± 0.00^c^ (+)	8.67 ± 0.33^d^ (+)	11.00 ± 0.58^c^ (+)	16.67 ± 0.88^d^ (++)
	16	13.67 ± 0.67^e^ (++)	16.00 ± 0.00^e^ (++)	17.33 ± 0.33^d^ (++)	19.33 ± 0.33^e^ (++)
Chl	30 (μg/disk)	21.33 ± 0.33^f^ (+++)	22.00 ± 0.00^f^ (+++)	24.67 ± 0.33^e^ (+++)	26.33 ± 0.33^f^ (+++)
MDM‐EUG	0	ND	ND	ND	ND
	1	1.67 ± 0.67^a^ (+)	2.00 ± 0.00^a^ (+)	2.67 ± 0.33^a^ (+)	3.00 ± 0.58^a^ (+)
	2	2.00 ± 0.00^ab^ (+)	2.33 ± 0.33^a^ (+)	3.00 ± 0.58^a^ (+)	3.67 ± 0.33^a^ (+)
	4	2.67 ± 0.33^ab^ (+)	3.33 ± 0.33^ab^ (+)	4.33 ± 0.67^ab^ (+)	5.67 ± 0.33^ab^ (+)
	8	7.33 ± 0.33^d^ (+)	9.67 ± 0.33^d^ (+)	10.00 ± 0.58^de^ (+)	11.00 ± 0.58^c^ (+)
	16	13.67 ± 0.67^f^ (++)	14.00 ± 0.00^e^ (++)	15.33 ± 0.33^f^ (++)	16.33 ± 0.33^de^ (++)
NMDM‐EUG	0	ND	ND	ND	ND
	1	3.33 ± 0.33^ab^ (+)	3.67 ± 0.33^ab^ (+)	4.00 ± 0.00^ab^ (+)	4.33 ± 0.33^a^ (+)
	2	4.00 ± 0.58^bc^ (+)	4.67 ± 0.33^bc^ (+)	5.67 ± 0.67^**bc**^ (+)	5.33 ± 0.33^ab^ (+)
	4	5.67 ± 0.33^cd^ (+)	6.33 ± 0.33^c^ (+)	7.67 ± 0.67^cd^ (+)	8.00 ± 0.58^b^ (+)
	8	10.00 ± 0.00^e^ (+)	11.33 ± 0.33^d^ (+)	12.00 ± 0.00^e^ (+)	13.67 ± 0.67^cd^ (++)
	16	15.33 ± 0.33^f^ (++)	16.33 ± 0.33^f^ (++)	17.67 ± 0.67^**f**^ (++)	18.00 ± 0.58^e^ (++)
Chl	30 (μg/disk)	21.67 ± 0.33^g^ (+++)	22.33 ± 0.67^g^ (+++)	24.00 ± 0.58^g^ (+++)	26.33 ± 0.88^f^ (+++)

*Note:* Values marked by different letters within the same columns for each antibacterial agent (ZEO or EUG separately) are significantly different according to Tukey's test (*p* ≤ 0.05). (+): Weak antibacterial activity (inhibitory zone ≤ 12 mm); (++): Moderate antibacterial activity (12 < inhibitory zone < 20 mm); (+++): Strong antibacterial activity (inhibitory zone ≥ 20 mm).

Abbreviations: Chl, chloramphenicol (positive control); MDM‐EUG, conventional mechanically deboned chicken meat protein coating‐forming solution containing eugenol; MDM‐ZEO, conventional mechanically deboned chicken meat protein coating‐forming solution containing *Z. clinopodioides* essential oil; ND, not detected; NMDM‐EUG, nanocomposite mechanically deboned chicken meat protein/nanoclay coating‐forming solution containing eugenol; NMDM‐ZEO, nanocomposite mechanically deboned chicken meat protein/nanoclay coating‐forming solution containing *Z. clinopodioides* essential oil.

The results indicated that gram‐positive bacteria were more sensitive to MDM and NMDM coatings containing ZEO or EUG compared to gram‐negative bacteria, which aligns with previous studies on the in vitro antibacterial effects of edible food packaging systems incorporating ZEO or EUG (Aminzare et al. 2022; Shahbazi 2018). The bilayer phospholipid membrane of gram‐positive bacteria cooperates with the hydrophobic components of these compounds, leading to cell demise by disrupting the bacterial enzyme system, leakage of vital cell components, and increasing ion permeability. Gram‐negative bacteria, on the other hand, show higher resistance to phenolic compounds because of the limited ability of hydrophobic substances to diffuse through their lipopolysaccharide membrane (Aminzare et al. 2022).

The findings also revealed that NMDM coatings with varying concentrations of ZEO or EUG exhibited larger inhibitory zones than MDM coatings with similar concentrations of bioactive compounds. In nanocomposite coatings, bioactive agents have an increased surface‐to‐volume ratio compared to conventional coatings, which increases the surface reactivity of antimicrobial agents at the nanoscale (Rehman et al. 2020). Additionally, synergistic antimicrobial effects between the bioactive compounds of EOs and nanoclay have been demonstrated. Nanoclay, by trapping bioactive compounds between its layers, protects them from premature evaporation and enhances their bactericidal effect (de Souza et al. 2020). Moreover, in nanocomposite systems, the surfactant released from the nanoclay layers outside the nanocomposite matrix supports bactericidal activities and creates a larger inhibition zone than conventional composites (Moustafa et al. 2023). In support of these results, previous studies have also demonstrated the in vitro antimicrobial activity of nanoclay‐based nanocomposite packaging systems, such as chitosan/carboxymethylcellulose films incorporating ZEO (Shahbazi 2018) and linear low density polyethylene films containing EUG (Tornuk et al. 2018) against foodborne pathogens.

### 3.4 Antibacterial Interactions Between ZEO and EUG in Conventional and Nanocomposite MDCM‐P Coatings

The results of the antibacterial interactions between ZEO and EUG in conventional and nanocomposite MDCM‐P coatings using the well‐diffusion method are shown in Tables 5 and 6. MDM and NMDM coatings containing 16 mg/mL of ZEO combined with 16 mg/mL of EUG showed significantly stronger antibacterial activity compared to other treatments against all examined bacteria (*p* ≤ 0.05). NMDM coatings containing the highest ZEO and EUG concentrations had strong antibacterial activity (inhibition zone ≥ 20 mm) against gram‐positive bacteria. Previous studies have reported that the combined use of plant‐derived antimicrobial compounds can lead to different types of interactions depending on factors such as bacterial species, compound ratio, and the physicochemical properties of the matrix (Aminzare et al. 2022). Other studies have also reported variable in vitro antibacterial interactions between ZEO (Shavisi, Basti, et al. 2017) or EUG (Aminzare et al. 2022) combined with other plant bioactive compounds in food packaging systems.

**TABLE 5:** Interaction antibacterial effects between ZEO and EUG in conventional MDCM‐P coatings using well‐diffusion method (Mean ± SE).

Bacteria	Inhibition zones (mm)			
	ZEO (8 mg/mL) + EUG (8 mg/mL)	ZEO (8 mg/mL) + EUG (16 mg/mL)	ZEO (16 mg/mL) + EUG (8 mg/mL)	ZEO (16 mg/mL) + EUG (16 mg/mL)
*E. coli* O157:H7	9.00 ± 0.00^a^ (+)	14.33 ± 0.33^b^ (++)	15.00 ± 0.00^b^ (++)	16.67 ± 0.33^c^ (++)
*S. enteritidis*	10.67 ± 0.67^a^ (+)	15.33 ± 0.33^b^ (++)	15.00 ± 0.00^b^ (++)	17.67 ± 0.67^c^ (++)
*S. aureus*	11.67 ± 0.67^a^ (+)	16.33 ± 0.33^b^ (++)	16.67 ± 0.33^b^ (++)	19.00 ± 0.58^c^ (++)
*L. monocytogenes*	12.00 ± 0.00^a^ (+)	17.67 ± 0.67^b^ (++)	17.33 ± 0.33^b^ (++)	19.67 ± 0.33^c^ (++)

*Note:* Values marked by different letters within the same rows are significantly different according to Tukey's test (*p* ≤ 0.05). (+): Weak antibacterial activity (inhibitory zone ≤ 12 mm); (++): Moderate antibacterial activity (12 mm < inhibitory zone < 20 mm).

**TABLE 6:** Interaction antibacterial effects between ZEO and EUG in nanocomposite MDCM‐P coatings using well‐diffusion method (Mean ± SE).

Bacteria	Inhibition zones (mm)			
	ZEO (8 mg/mL) + EUG (8 mg/mL)	ZEO (8 mg/mL) + EUG (16 mg/mL)	ZEO (16 mg/mL) + EUG (8 mg/mL)	ZEO (16 mg/mL) + EUG (16 mg/mL)
*E. coli* O157:H7	11.67 ± 0.67^a^ (+)	16.00 ± 0.00^b^ (++)	14.67 ± 0.33^b^ (++)	18.33 ± 0.33^c^ (++)
*S. enteritidis*	12.00 ± 0.00^a^ (+)	17.33 ± 0.33^b^ (++)	17.00 ± 0.00^b^ (++)	19.00 ± 0.58^c^ (++)
*S. aureus*	13.33 ± 0.33^a^ (++)	18.67 ± 0.33^b^ (++)	18.33 ± 0.33^b^ (++)	20.67 ± 0.33^c^ (+++)
*L. monocytogenes*	14.33 ± 0.33^a^ (++)	19.00 ± 0.58^b^ (++)	18.00 ± 0.00^b^ (++)	21.00 ± 0.58^c^ (+++)

*Note:* Values marked by different letters within the same rows are significantly different according to Tukey's test (*p* ≤ 0.05). (+): Weak antibacterial activity (inhibitory zone ≤ 12 mm); (++): Moderate antibacterial activity (12 < inhibitory zone < 20 mm); (+++): Strong antibacterial activity (inhibitory zone ≥ 20 mm).

### 3.5 Application of Conventional and Nanocomposite MDCM‐P Coatings Containing ZEO and EUG on Ostrich Meat

#### 3.5.1 Microbial Quality

**FIGURE 1:**
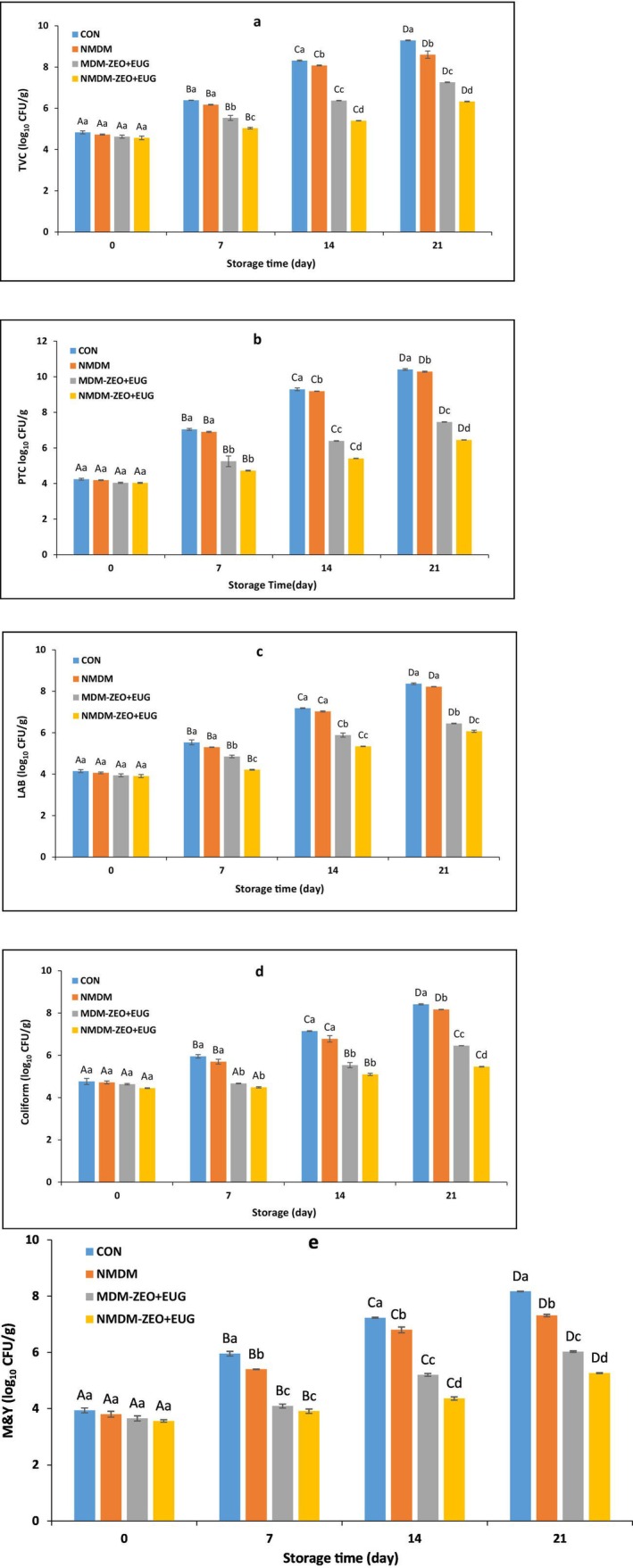
(a) Total viable count (TVC), (b) Psychrotrophic count (PTC), (c) Lactic acid bacteria (LAB), (d) Coliform bacteria, and (e) Molds and yeasts (M&Y) changes in ostrich meats treated with conventional and nanocomposite MDCM‐P coatings containing ZEO and EUG during 21 days of storage at 4°C (Mean ± SE). CON (control): Uncoated ostrich meat; NMDM: Ostrich meat treated with nanocomposite MDCM‐P/nanoclay coating without ZEO and EUG; MDM‐ZEO + EUG: Ostrich meat treated with conventional MDCM‐P coating containing ZEO (16 mg/mL) and EUG (16 mg/mL); NMDM‐ZEO + EUG: Ostrich meat treated with nanocomposite MDCM‐P/nanoclay coating containing ZEO (16 mg/mL) and EUG (16 mg/mL). Values marked by different capital letters within the same experimental group, as well as values marked with different lowercase letters within the same day, are significantly different according to Tukey's test (*p* ≤ 0.05).

The microbial population changes in ostrich meat treated with conventional and nanocomposite MDCM‐P coatings during 21 days of storage at 4°C are depicted in Figure 1. Total viable count (TVC) and psychrotrophic count (PTC) serve as key indicators for assessing the microbial quality of meat and its products (Saricaoglu and Turhan 2019). Figure 1a,b show that the initial TVC and PTC in the experimental groups were in the range of 4.57–4.83 and 4.04–4.24 log_10_ CFU/g, respectively (*p* ≥ 0.05), consistent with results from earlier studies (Heydari et al. 2020). However, significant upward trends in TVC and PTC were observed across all experimental groups over the storage time (*p* ≤ 0.05). At the end of the storage, the NMDM‐treated group exhibited significantly lower TVC and PTC levels than the CON group (*p* ≤ 0.05). This is probably due to the oxygen‐barrier features of the NMDM coating, aligning with previous research showing the inhibitory impact of edible coatings on total and psychrotrophic bacterial growth in ostrich meat (Heydari et al. 2020). Saricaoglu and Turhan (2019) reported that MDCM‐P coatings without antimicrobial agents were able to slow the growth of PTC and TVC in sujuk sausage compared to the uncoated control group during storage. Furthermore, nanoclay in nanocomposite films and coatings has been shown to enhance barrier properties against moisture and oxygen, thereby inhibiting bacterial growth in meat (Nath et al. 2022). Other studies indicate that the maximum permissible levels of TVC and PTC in fresh ostrich meat are considered to be 7 log_10_ CFU/g (Kiakojori et al. 2024). On the 14th day of storage, the TVC and PTC populations in the MDM‐ZEO + EUG and NMDM‐ZEO + EUG treatments did not reach the permissible limit, unlike the CON and NMDM groups. The antimicrobial properties of ZEO and EUG in the MDCM‐P coatings may account for this phenomenon. Supporting this claim, other studies have also reported the restricting effects of films and coatings containing ZEO or EUG in reducing the growth trends of TVC and PTC in various types of muscle foods (Hasan et al. 2019; Shavisi, Khanjari, et al. 2017; Yousefizadeh et al. 2022; Zhao et al. 2022). The highest antibacterial effect was observed in the NMDM‐ZEO + EUG treatment, with reductions of 2.96 and 3.96 log_10_ cycles for TVC and PTC, respectively, compared to the CON group (*p* ≤ 0.05). In the NMDM‐ZEO + EUG treatment, TVC and PTC levels remained the acceptable threshold even at the end of the storage period and showed a significant difference compared to the MDM‐ZEO + EUG group (*p* ≤ 0.05). This can be explained by the smaller droplet size of the antimicrobial bioactive compounds in the polymer matrix of the coating, which enhances their impact on the cell membrane and improves interaction with various molecular sites on the bacterial cell membrane (Ghaderi et al. 2024). Additionally, the presence of nanoclay can facilitate the sustained release of plant bioactive compounds from the nanocomposite coating and prevent their evaporation. As a result, nanocomposite coatings exhibit a longer antibacterial effect than conventional coatings (Nath et al. 2022). In support of these results, other studies have also highlighted the antimicrobial effects of nanoclay‐based active packaging containing ZEO or EUG on different meat products. For example, Kechagias et al. (2024) showed that low‐density polyethylene films incorporating EUG nanocarriers significantly reduced the TVC of fresh minced pork during 10 days of storage. Likewise, Khezrian and Shahbazi (2018) reported notable reductions in both TVC and PTC in minced camel meat wrapped with chitosan/carboxymethyl cellulose nanocomposite films containing ZEO over 14 days of storage.

Lactic acid bacteria (LAB) are a group of facultative anaerobic bacteria that make up a significant portion of the natural microflora in meat and are considered one of the primary contributors to the spoilage of packaged meat (Heydari‐Majd et al. 2019). The initial LAB population was in the range of 3.91–4.15 log_10_ CFU/g (Figure 1c), which was consistent with the results of previous studies (Juszczuk‐Kubiak et al. 2021). Over time, the LAB population increased significantly in all experimental groups (*p* ≤ 0.05). No significant difference was noted between the NMDM and CON groups by the end of the storage time (*p* ≥ 0.05). Ostrich meat coated with NMDM without bioactive compounds did not inhibit LAB growth, as LAB strains are facultative anaerobes that thrive in microaerophilic environments (Heydari‐Majd et al. 2019). Earlier studies suggest that the maximum acceptable levels of LAB population in some muscle foods stored at 4°C is 6 log_10_ CFU/g (Khezrian and Shahbazi 2018; Shafiei et al. 2024). The LAB population on day 14 of storage remained within the permissible limit only in the MDM‐ZEO + EUG and NMDM‐ZEO + EUG treatments, which can be due to the antimicrobial features of ZEO and EUG, as reported in earlier studies (Ekonomou et al. 2023; Hasan et al. 2019). NMDM‐ZEO + EUG treatment exhibited the lowest LAB population with reductions of 2.29 log_10_ cycles compared to the CON group (*p* ≤ 0.05). Similar studies have reported that nanocomposite coatings containing nanoclay and ZEO or EUG can effectively reduce LAB growth in muscle foods compared to untreated controls (Khezrian and Shahbazi 2018; Tornuk et al. 2015).

Coliform counts are commonly used to detect fecal contamination and serve as a hygienic indicator of the slaughter process (Shange 2020). As illustrated in Figure 1d, the initial coliform population ranged from 4.45 to 4.76 log_10_ CFU/g (*p* ≥ 0.05), aligning with the findings of previous studies (Mashak et al. 2019). Throughout the storage period, the coliform bacterial population increased significantly in all groups (*p* ≤ 0.05). According to Daniel et al. (2016) study, the acceptable limit for coliform bacteria in food is 6 log_10_ CFU/g. On day 14, coliform bacteria in the MDM‐ZEO + EUG and NMDM‐ZEO + EUG treatments remained below the permissible limit, likely due to the ZEO and EUG in the coatings. Supporting these results, previous studies have reported the anti‐coliform effects of *Ziziphora tenuior* EO (Mahdavi and Nobakht 2018). Additionally, Zhang et al. (2013) found that soy protein isolate‐based edible films containing EUG reduced the coliform population in pork stored at refrigeration temperature. NMDM‐ZEO + EUG treatment showed the highest antibacterial effect with reductions of 2.95 log_10_ cycle compared to the CON group (*p* ≤ 0.05), keeping bacterial growth within the permissible limit. This is likely due to the longer‐lasting effects of nanocomposite coatings containing nanoclay and bioactive compounds. Consistent with our findings, Pires et al. (2018) reported that chitosan/montmorillonite bionanocomposites containing rosemary and ginger EOs effectively limited the growth of coliforms in fresh poultry meat, attributing this effect to the enhanced antimicrobial performance of chitosan in the presence of nanoclay.

Molds and yeasts (M&Y) play a significant role in meat spoilage and are useful indicators of meat quality. Fungi contaminate meat through the production of mycotoxins, which can lead to liver damage and food poisoning in humans (Zakki et al. 2017). As depicted in Figure 1e, the initial M&Y population in all experimental groups ranged from 3.56–3.94 log_10_ CFU/g (*p* ≥ 0.05). These differences are likely attributable to variations in slaughterhouse hygiene, meat processing conditions, and the time elapsed between slaughter and sampling (Zakki et al. 2017). Meat contamination with M&Y primarily occurs during handling (Hosseini et al. 2021). The M&Y population significantly increased in all groups during storage (*p* ≤ 0.05). By the completion of the storage period, the NMDM coating reduced fungal growth compared to the CON group, likely due to its oxygen barrier properties, aligning with previous studies (Heydari et al. 2020). On this day, the M&Y population in ostrich meat treated with MDM‐ZEO + EUG and NMDM‐ZEO + EUG was notably lower in comparison to other groups (*p* ≤ 0.05). Supporting these findings, previous studies have reported the antifungal effects of edible coatings encompassing ZEO on chicken fillets (Hamedi et al. 2017) and clove EO (with EUG as its main component) on chicken breast meat (Hosseini et al. 2021). The lowest M&Y population was observed in the NMDM‐ZEO + EUG treatment, with reductions of 2.91 log_10_ cycle compared to the CON group (*p* ≤ 0.05). These results are consistent with the study by Tornuk et al. (2015), which reported the antifungal effects of nanocomposite films containing nanoclay and phenolic bioactive compounds (eugenol, thymol, and carvacrol) on the M&Y population in beef.

#### 3.5.2 Microbial Safety

**FIGURE 2:**
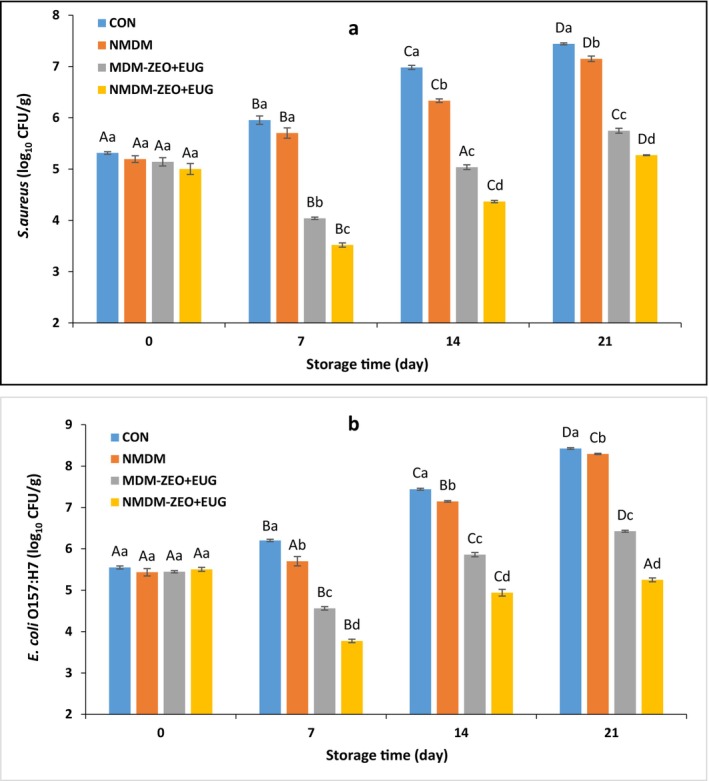
(a) *S. aureus* and (b) *E. coli* O157:H7 populations in ostrich meats treated with conventional and nanocomposite MDCM‐P coatings containing ZEO and EUG during 21 days of storage at 4°C (mean ± SE). CON (control): Uncoated ostrich meat; NMDM: Ostrich meat treated with nanocomposite MDCM‐P/nanoclay coating without ZEO and EUG; MDM‐ZEO + EUG: Ostrich meat treated with conventional MDCM‐P coating containing ZEO (16 mg/mL) and EUG (16 mg/mL); NMDM‐ZEO + EUG: Ostrich meat treated with nanocomposite MDCM‐P/nanoclay coating containing ZEO (16 mg/mL) and EUG (16 mg/mL). Values marked by different capital letters within the same experimental group, as well as values marked with different lowercase letters within the same day, are significantly different according to Tukey's test (*p* ≤ 0.05).

The microbial safety of ostrich meat samples was evaluated through inoculation with *S. aureus* and *E. coli* O157:H7 pathogenic bacteria in ostrich meat. Figure 2a,b show the growth trends of *S. aureus* and *E. coli* O157:H7 in ostrich meat treated with conventional and nanocomposite MDCM‐P coatings containing ZEO and EUG during 21 days of storage at 4°C, respectively. The initial populations of *E. coli* O157:H7 and *S. aureus* ranged from 5.43 to 5.55 and from 5 to 5.31 log_10_ CFU/g (*p* ≥ 0.05). During storage, bacterial growth in the CON and NMDM groups increased and peaked on day 21 (*p* ≤ 0.05). In contrast, the bacterial populations in the MDM‐ZEO + EUG and NMDM‐ZEO + EUG treatments decreased throughout the first 7 days and then increased until the end of the storage period, which confirms the antibacterial effects of ZEO and EUG in the coatings. The NMDM‐ZEO + EUG treatment had the strongest antibacterial effect, reducing *S. aureus* and *E. coli* O157:H7 populations by 2.17 and 3.18 log_10_ cycles, respectively, compared to the CON group (*p* ≤ 0.05). In line with these results, Saricaoglu and Turhan (2019) attributed the inhibitory effects of MDCM‐P coatings containing clove EO against *S. aureus* in beef sucuks to the EUG present in the EO. In a similar study, Shahbazi et al. (2016) found the inhibitory effects of ZEO on the growth of *S. aureus* inoculated in beef patty during refrigerated storage. Furthermore, other studies have also reported the inhibitory effects of various active nanocomposite packaging systems containing nanoclay and either ZEO or EUG against *E. coli* O157:H7 inoculated in different muscle foods (Khezrian and Shahbazi 2018; Tornuk et al. 2015).

#### 3.5.3 Sensory Characteristics

**FIGURE 3:**
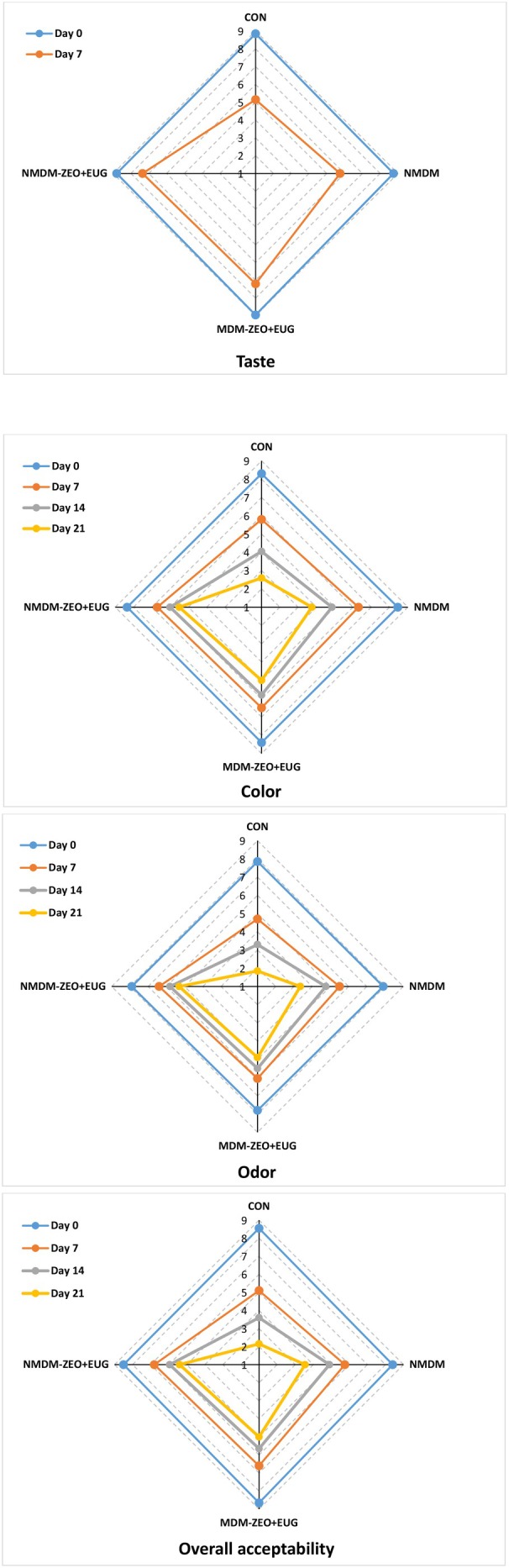
Sensory properties of ostrich meats treated with conventional and nanocomposite MDCM‐P coatings containing ZEO and EUG during 21 days of storage at 4°C. CON (control): Uncoated ostrich meat; NMDM: Ostrich meat treated with nanocomposite MDCM‐P/nanoclay coating without ZEO and EUG; MDM‐ZEO + EUG: Ostrich meat treated with conventional MDCM‐P coating containing ZEO (16 mg/mL) and EUG (16 mg/mL); NMDM‐ZEO + EUG: Ostrich meat treated with nanocomposite MDCM‐P/nanoclay coating containing ZEO (16 mg/mL) and EUG (16 mg/mL).

It is essential to evaluate the potential impact of natural preservatives on sensory characteristics when enhancing the quality of meat products (Lamri et al. 2021). Changes in the sensory characteristics of ostrich meat during 21 days of storage are presented in Figure 3. As illustrated, the scores assigned to all experimental groups for all sensory attributes decreased significantly over the storage period (*p* ≤ 0.05). However, signs of microbial spoilage, including a gel‐like texture, apparent color change, and unpleasant smell, appeared more rapidly in the CON sample than in the other sample groups (*p* ≤ 0.05). The growth and activity of spoilage microorganisms in ostrich meat can produce undesirable flavors, odors, and unnatural colors, often associated with microbial metabolites and their enzymatic reactions (Sajadi et al. 2024). Simultaneously, the MDM‐ZEO + EUG and NMDM‐ZEO + EUG treatments obtained higher scores than the other experimental groups (scores higher than 4 in all sensory characteristics), indicating the inhibitory effects of ZEO and EUG against spoilage microorganisms and consequently retarding the development of undesirable sensory properties in ostrich meat. Similar studies have shown that edible coatings with ZEO or EUG are effective in preventing microbial spoilage, thereby enhancing the sensory properties of various types of meat (Hasan et al. 2019; Shavisi, Khanjari, et al. 2017; Yousefizadeh et al. 2022). The NMDM‐ZEO + EUG treatment achieved the highest overall acceptability scores, likely due to the more stable microbial suppression effects of the nanocomposite coating than the conventional coating. Additionally, since the strong flavor of plant EOs can adversely affect meat's sensory characteristics (Lamri et al. 2021), nanosynthesis of EOs and incorporating nanoclay into the nanocomposite coating can control the release of aromatic components and enhance the product's sensory quality (Cheikh et al. 2022). In support of this claim, Khezrian and Shahbazi (2018) reported that chitosan and carboxymethyl cellulose nanocomposite films containing nanoclay and ZEO enhanced the sensory features of camel meat.

## 4 Conclusion

This study demonstrated that incorporating ZEO and EUG into both conventional and nanocomposite (with nanoclay) MDCM‐P coatings enhanced their in vitro antibacterial activity against two gram‐positive ( *S. aureus* and *L. monocytogenes* ) and two gram‐negative ( *E. coli* O157:H7 and *S. enteritidis* ) bacteria. Antibacterial effects increased with increasing ZEO or EUG concentrations. Gram‐positive bacteria exhibited greater sensitivity than gram‐negative bacteria, with the strongest antibacterial effect observed against *L. monocytogenes* . The combined use of ZEO and EUG in the MDCM‐P coatings exhibited stronger antibacterial properties than their individual applications. Furthermore, nanocomposite coatings demonstrated superior antibacterial activity compared to conventional coatings at equivalent concentrations of ZEO and EUG. Based on microbiological (TVC ≤ 7 log_10_ CFU/g) and sensory (overall acceptability score ≥ 4) criteria, the shelf‐life of refrigerated ostrich meat was determined to be 7 days for the CON and NMDM samples. The MDM‐ZEO + EUG treatment extended the shelf‐life to 14 days, while the NMDM‐ZEO + EUG coating maintained acceptable microbiological and sensory quality throughout the 21‐day storage period, extending the shelf‐life by at least 14 days compared to the control. Additionally, the NMDM‐ZEO + EUG coating effectively inhibited the growth of inoculated *E. coli* O157:H7 and *S. aureus* during storage. These results indicate that NMDM‐ZEO + EUG is a potential active packaging material for enhancing the microbial quality preservation and microbial safety of refrigerated ostrich meat.

## Author Contributions

**Hassan Barkhordari:** conceptualization, investigation, writing – original draft, formal analysis, data curation, resources, methodology, funding acquisition. **Majid Aminzare:** conceptualization, data curation, formal analysis, funding acquisition, investigation, methodology, project administration, resources, software, supervision, validation, visualization, writing – original draft, writing – review and editing. **Hassan Hassanzadazar:** conceptualization, formal analysis, investigation, methodology, project administration, resources, supervision, validation, writing – review and editing. **Adel Mirza Alizadeh:** conceptualization, writing – review and editing, validation, visualization, methodology. **Mahsa Hashemi:** conceptualization, investigation, writing – review and editing, visualization, validation, methodology, data curation, supervision. **Reza Tahergorabi:** conceptualization, formal analysis, investigation, methodology, validation, writing – review and editing. **Shahin Roohinejad:** conceptualization, investigation, methodology, validation, writing – review and editing.

## Funding

This work was supported by Zanjan University of Medical Sciences (A‐12‐964‐20).

## Ethics Statement

Informed consent was obtained from all participants in the sensory evaluation and the study was approved by the Ethics Committee of Zanjan University of Medical Sciences (Ethical Code: IR.ZUMS.BLC.1401.053).

## Conflicts of Interest

The authors declare no conflicts of interest.

## Data Availability

The data that support the findings of this study are available from the corresponding author upon reasonable request.
